# RNA aptamers with specific binding affinity to CD40 (CD40Apt) represents a promising antagonist of the CD40-CD40L signaling for thyroid-associated ophthalmopathy (TAO) treatment in mouse

**DOI:** 10.1186/s12967-023-04217-0

**Published:** 2023-06-18

**Authors:** Yizhi Chen, Renhong Tang, Wei Xiong, Feng Zhang, Nuo Wang, Bingyu Xie, Jiamin Cao, Zhuokun Chen, Chen Ma

**Affiliations:** Department of Ophthalmology, Third Xiangya Hospital, Central South University, Changsha, China

**Keywords:** CD40 aptamer (CD40Apt), Thyroid-associated ophthalmopathy (TAO), The CD40-CD40L signaling, Orbital fibroblast activation

## Abstract

Thyroid-associated ophthalmopathy (TAO) is the most common autoimmune inflammatory diseases of the orbit. The CD40-CD40L pathway has been regarded as a potential molecular mechanism contributing to the development and progression of TAO, and RNA aptamers with specific binding affinity to CD40 (CD40Apt) represents a promising inhibitor of the CD40-CD40L signaling in TAO treatment. In this study, CD40Apt was confirmed to specifically recognize mouse CD40-positive ortibtal fibroblast. Mouse orbital fibroblasts were isolated from TAO mice model orbital tissues and validated. In TGF-β-induced orbital fibroblast activation model in vitro, CD40Apt administration inhibited TGF-β-induced cell viability, decreased TGF-β-induced α-SMA, Collagen I, Timp-1, and vimentin levels, and suppressed TGF-β-induced phosphorylation of Erk, p38, JNK, and NF-κB. In TAO mice model in vivo, CD40Apt caused no significant differences to the body weight of mice; furthermore, CD40Apt improved the eyelid broadening, ameliorated inflammatory infiltration and the hyperplasia in orbital muscle and adipose tissues in model mice. Concerning orbital fibroblast activation, CD40Apt reduced the levels of CD40, collagen I, TGF-β, and α-SMA in orbital muscle and adipose tissues of model mice. Finally, CD40Apt administration significantly suppressed Erk, p38, JNK, and NF-κB phosphorylation. In conclusion, CD40Apt, specifically binds to CD40 proteins in their natural state on the cell surface with high affinity, could suppress mouse orbital fibroblast activation, therefore improving TAO in mice model through the CD40 and downstream signaling pathways. CD40Apt represents a promising antagonist of the CD40-CD40L signaling for TAO treatment.

**Supplementary Information:**

The online version contains supplementary material available at 10.1186/s12967-023-04217-0.

## Introduction

Thyroid-associated ophthalmopathy (TAO), also referred to as Graves’ ophthalmopathy (GO), is the most common autoimmune inflammatory diseases of the orbit [1, 2]. From mild discomfort to severe expansion of extraocular muscles, proptosis, ophthalmoplegia, exposure keratopathy, and optic neuropathy, TAO exhibits a wide range of clinical severity. Most orbital problems in patients with moderate to severe TAO were caused by edematous changes of connective tissue and extraocular muscles [3], which is caused by extracellular accumulation of hyaluronic acid [4].

TAO is associated with the aberrant inflammatory cytokine secretion [5, 6]. These overexpressed inflammatory cytokines enhanced thyroid lymphocyte infiltration and B-cell activation, resulting in the synthesis of autoimmune antibodies against thyroid antigens during the progression of TAO [7]. Fibroblasts are critical effector cells involved in tissue healing, inflammatory process, disease development and immune function [8]. Concerning TAO, resident orbital fibroblasts and fibrocytes that infiltrate TAO patients’ orbit play a crucial mediatory role in the pathology of diseases [9, 10]. In response to proinflammatory cytokines, TAO patients’ orbital fibroblasts led to excessive production of hyaluronic acid, glycosaminoglycan and inflammatory cytokines including interleukin IL-6 and IL-8 [11, 12]. TAO patients’ orbital fibroblasts induce a higher rate of proliferation and are more prone to adipocyte differentiation as compared to those from control subjects, in turn, accelerate TAO progression [13].

Recently, the relationship between the CD40-CD40L pathway and TAO has emerged as a potential molecular mechanism in the development and progression of TAO. CD40 is highly expressed in orbital fibroblasts, and CD40-positive fibroblasts are significantly more abundant in the orbital tissues of TAO patients than in healthy subjects. CD40L induces orbital fibroblasts to produce many inflammatory cytokines such as IL-6, IL-8, COX-2, and PGE2 and synthesize hyaluronic acid [14]. Feldon et al. [15] demonstrated the involvement of T cells and orbital fibroblasts in an antigen-dependent positive feedback loop where autoantigen presentation by fibroblasts through MHC class II and CD40-CD40L ligand leads to the activation of T cells; these activated T cells stimulate fibroblasts to proliferate, resulting in fibroblast-related disorders in TAO. The CD40-CD40L signaling pathway can also activate NF-κB to undergo significant nuclear translocation and initiate transcription of inflammation-associated genes such as IL-6 and IL-8 [16]. Therefore, blocking the CD40-CD40L signaling pathway may reduce the degree of orbital fibroblasts activation, thereby slowing or even inhibiting TAO development.

Aptamers, synthetically created oligonucleotides which have abilities to bind with high affinity and specificity to a wide range of small target molecules, have proven to be a viable alternative to mAbs in various biological processes [17, 18]. Depending on its sequencing, each RNA or single-strand DNA molecule might have a unique 3D structure. A unique molecular structure that can bind to the ligand with great affinity and specificity is produced by this folding arrangement. Due to this characteristic, aptamers may bind target molecules with high specificity and affinity higher than that of corresponding antibodies [19, 20]. Aptamers have reduced immunogenicity than antibodies. Aptamers can be produced by chemical synthesis and are not cell-based products, which greatly simplifies GMP manufacturing required for clinical trials. In the event of negative effects, their action can also be swiftly stopped by an antidote [21, 22]. As a result, aptamers have become more effective in the field of medicine. RNA aptamers with specific binding affinity to CD40 (CD40Apt) have been developed [23]; considering the advantages of aptamers, CD40Apt represent promising inhibitor of the CD40-CD40L signaling in TAO treatment.

In this study, the affinity and specificity of CD40Apt were verified. Mouse orbital fibroblasts were isolated and used for the activation model; the effects of CD40Apt on orbital fibroblast activation were investigated. In orbital fibroblast activation model, the effects of CD40Apt on the CD40 signaling were investigated. Mouse model of TAO was established by TSHR adenovirus challenge and verified, the effects of CD40Apt intervention on the progression and severity of TAO in mouse, as well as the CD40 signaling were investigated.

## Materials and methods

### CD40 aptamer binding specificity and affinity

The CD40 aptamer sequences were reported as previous description [23] with 2-Fluoro-UTP and 2-Fluoro-CTP modification and generated by General Biol (Chuzhou, China). Control vector (NC), CD40-overexpressing vector (CD40), and control short hairpin RNA (sh-NC), sh-CD40 were commercially acquired from Genepharma (Shanghai, China) and transfected into 293 T cells or TAO mouse orbital fibroblasts with Lipofectamine 3000 reagent, respectively. Normal and transfected TAO mouse orbital fibroblasts or 293T cells were blocked with ice-cold PBS plus 100 µg/mL salmon sperm DNA and 1% BSA, and rinsed two times with a binding buffer. Next, cells were incubated with different concentration (50, 100, 150, 200, 250, 500 and 750 nM) of FAM-labelled CD40 aptamer in binding buffer at room temperature. Cells were then rinsed thrice with binding buffer. Cells were resuspended in 200 µL of binding buffer prior to flow cytometry assay (Novocyte, China). To calculate the MFI (mean fluorescence intensity) of specific binding, the MFI values of the untreated cells were subtracted from the MFI values obtained from aptamer-treated cells. The Equilibrium dissociation constants (Kd) value was calculated with the equation Y = Bmax X/(K_D_ + X) using the GraphPad Prism 6.0 software.

### Aptamer-Mediated pull-down assay

Orbital fibroblasts were rinsed thrice with precooling DPBS before being lysed at 4 °C for 30 min in 5 mL hypotonic buffer (washing buffer supplemented with 10× cocktail, 1 M Tris–HCl, and 100× PMSF). Following centrifugation, the debris was rinsed thrice using 5 mL hypotonic buffer and dissolved at 4 °C for 30 min in 2 mL lysis buffer (hypotonic buffer added with 1% Triton X-100). The supernatant was harvested and blocked with binding buffer contained with 20% FBS and 0.1 mg/ml salmon sperm DNA for 1 h and then followed by 1-h incubation at 4 °C with 150 pmol of non-binding biotin-labeled library sequences as a non-specific competitor or 150 pmol biotin-labeled CD40Apt. The protein-aptamer complexes were underwent 1-h incubation with 200 μl of streptavidin-coated magnetic beads (Beyotime, China) at 4 °C with rotation. The beads were collected onto a magnetic stand, then the protein-RNA complex was retained. The harvested magnetic beads were washed thrice with DPBS. The beads and the captured proteins were eluted by boiling in 30 μL loading buffer and separated using PAGE (12%, SDS-PAGE). The captured proteins were detected by immunoblotting using specific CD40 antibody.

### RNA stability in serum

To investigate the stability of CD40 aptamer against nucleases, 5 µL of aptamers (10 µM) were mixed with 5 µL of mouse serum and incubated at 37 ℃ for 0 h, 2 h, 4 h, 8 h, 24 h, 48 h, 4 days, 7 days and 14 days, respectively. After incubation for the assigned time interval, a 50 μl sample was removed and immediately placed in a − 80 °C freezer for storage until all samples were harvested. Each sample was electrophoresed on a 12% native PAGE and checked using a Bio-rad gel imaging system. Three independent experiments were performed.

### Immunization of BALB/c mouse with TSHR-adenovirus

The construction, amplification, purification of non-replicative recombinant adenovirus expressing the human TSHR A subunit (TSHR-adenovirus, Ad-TSHR) and the negative control (Ad-NC) and determination of the viral particle concentration were carried out following the methods documented previously [24, 25]. Thirty BALB/c mice were randomly assigned into five groups: Control group (n = 6), TAO model group (n = 6), TAO + PBS group (n = 6), TAO + Apt-control group (n = 6) and TAO + CD40Apt group (n = 6). The experimental group was subjected to intramuscular injection with TSR-adenovirus (PSB1317, Ori-bio, Changsha, China), the control group was subjected to injection with empty-loaded adenovirus (PMT407, Ori-bio), and the blank group were subjected to injection with PBS. Mice were kept under conventional breeding environment in 12 h light:12 h dark cycle, during which all mice had free access to normal chow diet and water.

For TAO model establishment, intramuscular injection was performed every 3 weeks, i.e., 0, 3, 6, 9, 12, 15, 18, 21, and 24 weeks, every Thursday between 15:00 to 16:00. Before injection, the mice were anesthetized with chloral hydrate by intraperitoneal administration. The titers of Ad-TSHR and Ad-NC were 2.97 × 10^10^ and 6.20 × 10^10^. Mouse femur muscle on the left hind leg was found and marked. After inserting the needle 0.5 mm vertically to the marked position, when it reaches the depth of the femoral muscle, 50μL of adenovirus solution was injected at a time. As for aptamer administration, mice were subjected to periorbital injection of 200 pmol of either CD40Apt-dimer, Apt-control (in 50 μl), or an equivalent volume of PBS at week 12 to week 24 twice per week. The schematic diagram of animal experimental treatment process is shown in Additional file 1: Fig. S1. All experiments were carried out in compliance with the principles and methods stated in the Guideline for the Care and Use of Laboratory Animals in the Third Xiangya Hospital.

The appearance of mice eyes was observed and photographed. The body weight of mice was recorded once a week. After mice were sacrificed under anesthesia, the serum was collected for ELISA and the orbital adipose and fibrous tissues were harvested for histopathological examination and primary orbital fibroblasts isolation.

### Primary orbital fibroblasts isolation from TAO mice

The mouse orbital tissues attached to plastic culture dishes and were covered with medium Resulting fibroblast monolayers were serially passaged with gentle trypsin/EDTA digestion. Isolated mouse orbital fibroblasts were cultivated in DMEM supplemented with 10% FBS and antibiotics.

### Cell culture and treatment

293T cell line was collected from ATCC (Manassas, VA, USA) and cultivated in Eagle's Minimum Essential Medium (EMEM, 30-2003, ATCC) supplemented with 10% FBS. For orbital fibroblasts activation, mouse TAO orbital fibroblasts was treated with 10 ng/ml TGF-β for 48 h. For CD40Apt incubation, orbital fibroblasts were incubated with 100, 250, 500 and 750 nM CD40Apt for 24, 48, 72 h or incubated with 500 nM CD40Apt for 48 h.

### Immunofluorescent staining (IF)

Isolated orbital fibroblasts were collected, fixed in 4% paraformaldehyde, and then blocked with goat serum (5%) for 30 min at RT, followed by an overnight incubation at 4 °C with primary anti-α-SMA (55135-1-AP, Proteintech, Wuhan, China). Slides were washed thrice in phosphate-buffered saline (PBS) added with 0.1% Tween 20, followed by 1-h incubation with secondary antibodies at RT at dark. After 10-min nuclear staining with DAPI (cat #P36931; Life Technologies), the commercial mounting media (Vectashield; Vector Laboratories, Burlingame, CA) was employed to mount cells and a fluorescent microscope was applied to examine cells.

### CCK-8

A Cell Counting Kit-8 (CCK-8) assay kit was employed to assess cell viability. At the designated time point, CCK-8 solution at a 1:10 dilution was added to the media, and the cells were cultivated for another 4 h. Next, a multi-function microplate reader (Bio-rad, USA) was utilized to detect the OD value of each well at the wavelength of 450 nm.

### qRT-PCR

RNA was extracted by TRIzol and reverse transcribed to cDNA using cDNA synthesis kit (Genstar, China). The SYBR Green PCR Master Mix (Qiagen, Germany) was used on a 7500 Real Time PCR System (Applied Biosystems, USA) to amplify RNA. The 2^−ΔΔct^ method was applied to calculate the RNA expression of each sample [26].

### Immunoblotting

RIPA Buffer (Tris–HCl 25 mM pH 7.4, NaCl 150 mM, 1% Triton X-100, Sodium Deoxycholate 1%, SDS 0.1%, EDTA 2 mM) containing a protease/phosphatase inhibitor mixture (Roche) was utlized to lyse cells. After electrophoresis with SDS-PAGE, the separated proteins from the gel (50 μg) were transferred on nitrocellulose (NC) membranes, followed by incubation with primary antibodies. Following incubation with appropriate HRP-labeled secondary antibodies, ECL HRP substrate (Beyotime) was employed to detect signals. The primary antibodies used were as follow: α-SMA (55135-1-AP, Proteintech), Collagen I (14695-1-AP, Proteintech), Timp-1 (CSB-PA023560YA01HU, CUSABIO, Wuhan, China), Vimentin (10366-1-AP, Proteintech), Erk (16443-1-AP, Proteintech), p-Erk (sc-81492, Santa Cruz Biotechnology, Santa Cruz, CA, USA), p38 (14064-1-AP, Proteintech), p-p38 (AF4001, Affinity, Changzhou, China), JNK (AF6319, Affinity), p-JNK (AF3318, Affinity), NF-κB (AF5006, Affinity), p-NF-κB (AF2006, Affinity).

### ELISA

The concentrations of mouse serum T4, TSH, and TRAb were examined by ELISA with corresponding ELISA kit (RXJ202844M for T4, RX203002M for TSH and RX202225M for TRAb) were obtained from Ruixin Biotech (Quanzhou, China) as directed by manufacturer-supplied protocols.

### Histopathological examination

Mouse orbital adipose and fibrous tissues were applied for histopathological examinations. Tissue samples were embedded in paraffin wax to prepare 5-μm-thick slices followed by H&E or Masson staining. After the staining, tissue sections were soaked in gradient ethanol concentration, 5 min for each concentration, soaked in xylene, and sealed with gum. A light microscope (Olympus, Tokyo, Japan) was employed to examine stained slices for histopathological analysis.

### IHC staining

Dewaxed and rehydrated orbital muscle and adipose tissue sections were quenched with 3% hydrogen peroxide solution for 10 min to inhibit the activity of endogenous peroxidase, and then rinsed with distilled water and PBS added with 0.15 M NaCl and 4% BSA. The nonspecific binding sites for immunoglobulins were closed via 30-min incubation with 5% normal goat serum in PBS added with 1% BSA, followed by incubation with antibodies against CD40 (AF5336, Affinity), collagen I (14695-1-AP, Proteintech), TGF-β1 (GB11179, Servicebio, China), or α-SMA (55135-1-AP, Proteintech) at 4 °C overnight. Sections were incubated with diluted goat anti-rabbit IgG (Vector Laboratories, Burlingame, CA) for 30 min. After washing with PBS-BSA for 15 min, the Vecta-lab “Elite” (ABC) kit (Vector Laboratories) was employed to obtain immunohistochemical visualization. After 5-min incubation with 0.05 M Tris buffer, pH 7.2 with 0.01% H_2_O_2_ and 0.05% diaminobenzidine tetrahydrochloride (DAB) (Sigma-Aldrich, St. Louis, MO, USA) to show brown immunoreactive cells, peroxidase activity was observed. Lastly, sections were counterstained with hematoxylin, dehydrated and sealed.

### Statistical analysis

All data are expressed in terms of mean ± standard deviation (SD). Comparisons among groups were assessed using one-way ANOVA followed Tukey’s multiple comparisons test. Comparisons between groups were assessed using a Student’s *t*-test. The significance level was set at *P* < 0.05.

## Results

### The binding affinity and specificity of aptamer to CD40

**Fig. 1:**
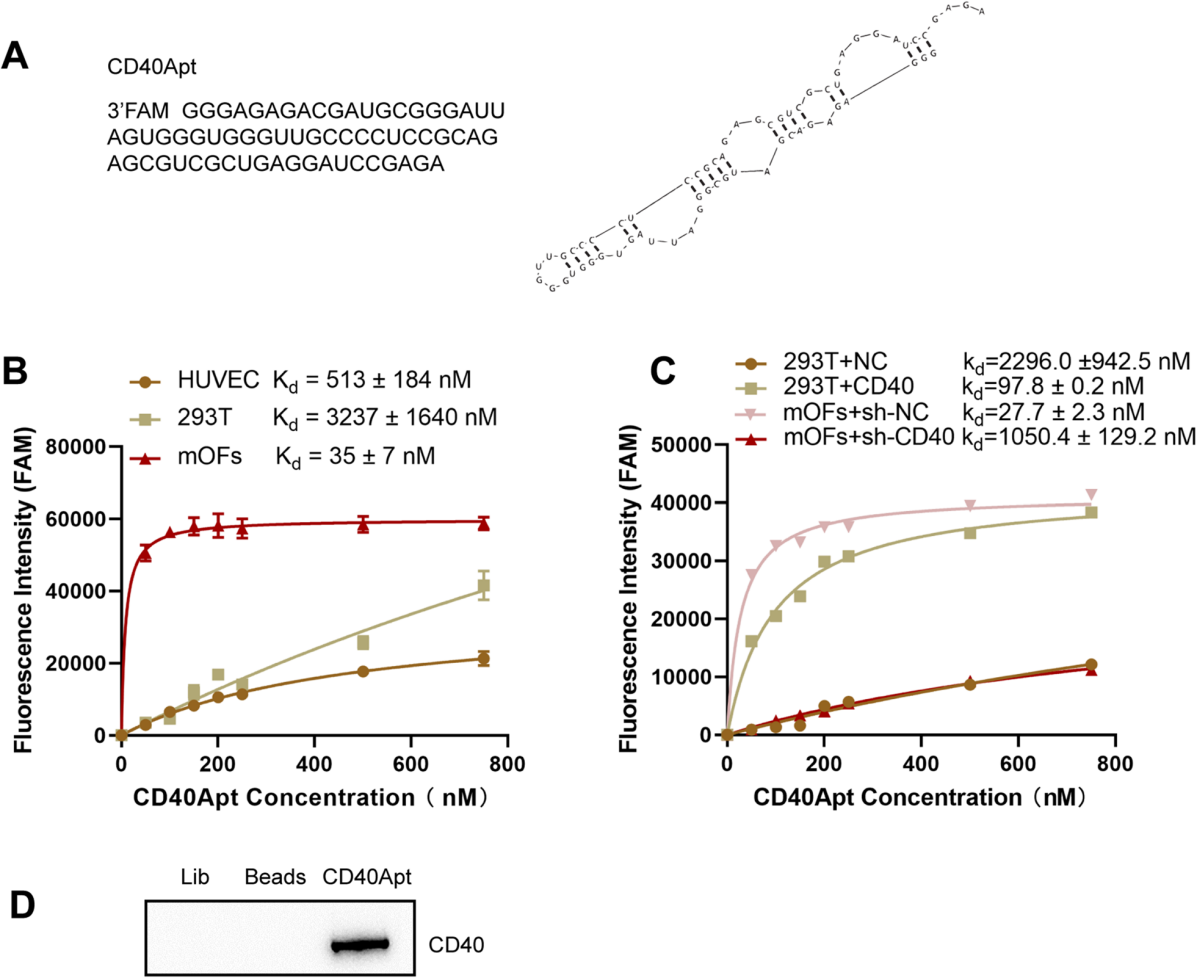
The binding affinity and specificity of aptamer to CD40. **A** The predicted secondary structure of CD40 aptamer (CD40Apt) using mfold program. **B** Equilibrium dissociation constants (Kd) of CD40Apt to TAO mouse orbital fibroblasts, 293 T and HUVEC were detected by Flow cytometry. **C** Kd in CD40 knocked-down TAO mouse orbital fibroblasts and CD40 overexpressed 293T were detected by Flow cytometry. **D** Aptamer pull-down assay was applied to detect the binding between CD40Apt and CD40 in mouse orbital fibroblasts

The secondary structure of CD40Apt was predicted using mfold program and shown in Fig. 1A. For diagnostic and targeted therapeutic uses, CD40Apt is required to target mouse CD40 protein molecules in their natural state on the cell surface. To that purpose, the ability of CD40Apt to bind to mouse CD40-positive cells was determined. To begin, FAM-tagged CD40Apt was synthesized, followed by 1-h incubation at 4 °C with CD40-negative HEK293T cells, human CD40-postive HUVECs, and TAO mouse isolated orbital fibroblasts (orbital fibroblasts, CD40 positive), Next, flow cytometry assay was conducted. Based on kinetic assays, the CD40Apt bound with high affinity to the tested mouse CD40 expressing cells, with dissociation equilibrium constants of 35 nM for TAO mouse orbital fibroblasts (Fig. 1B). Besides, the effects of overexpression of CD40 on the affinity of CD40Apt with HEK293T cells and knockdown of CD40 on the affinity of CD40Apt with TAO mouse orbital fibroblasts were investigated. The results indicated that overexpression of CD40 promoted the affinity of CD40Apt with HEK293T cells and knockdown of CD40 reduced the affinity of CD40Apt with TAO mouse orbital fibroblasts (Fig. 1C). Moreover, aptamer pull-down assay was carried out to validate the binding between CD40Apt and CD40 in TAO mouse orbital fibroblasts. Figure 1D shows that compared with negative control beads, cellular CD40 protein could be pulled down by CD40Apt. Therefore, CD40Apt could specifically recognize CD40-positive cells and bind to CD40 protein.

### Effects of CD40Apt on TGF-β-induced orbital fibroblast activation

Before investigating the effects of CD40Apt on the activation of orbital fibroblasts, orbital fibroblasts were isolated from TAO mouse orbital tissues and treated with 10 ng/ml TGF-β for 48 h to simulate its natural activated status. The levels of orbital fibroblast marker α-SMA were examined using IF staining; Additional file 2: Fig. S2A shows that orbital fibroblasts expressed higher α-SMA compared with normal orbital fibroblasts (NOFs). Regarding cell proliferation, TGF-β stimulation significantly promoted cell viability of orbital fibroblasts (Additional file 2: Fig. S2B). Concerning fibroblast activation, TGF-β stimulation significantly upregulated α-SMA, collagen I, Timp-1, and vimentin mRNA expression levels (Additional file 2: Fig. S2C), and remarkably increased α-SMA, collagen I, Timp-1, and vimentin protein contents (Additional file 2: Fig. S2D). Consistently, the levels of collagen I in supernatant were also elevated by TGF-β stimulation (Additional file 2: Fig. S2E). These findings suggest that orbital fibroblasts were successfully isolated and activated by TGF-β stimulation.

**Fig. 2:**
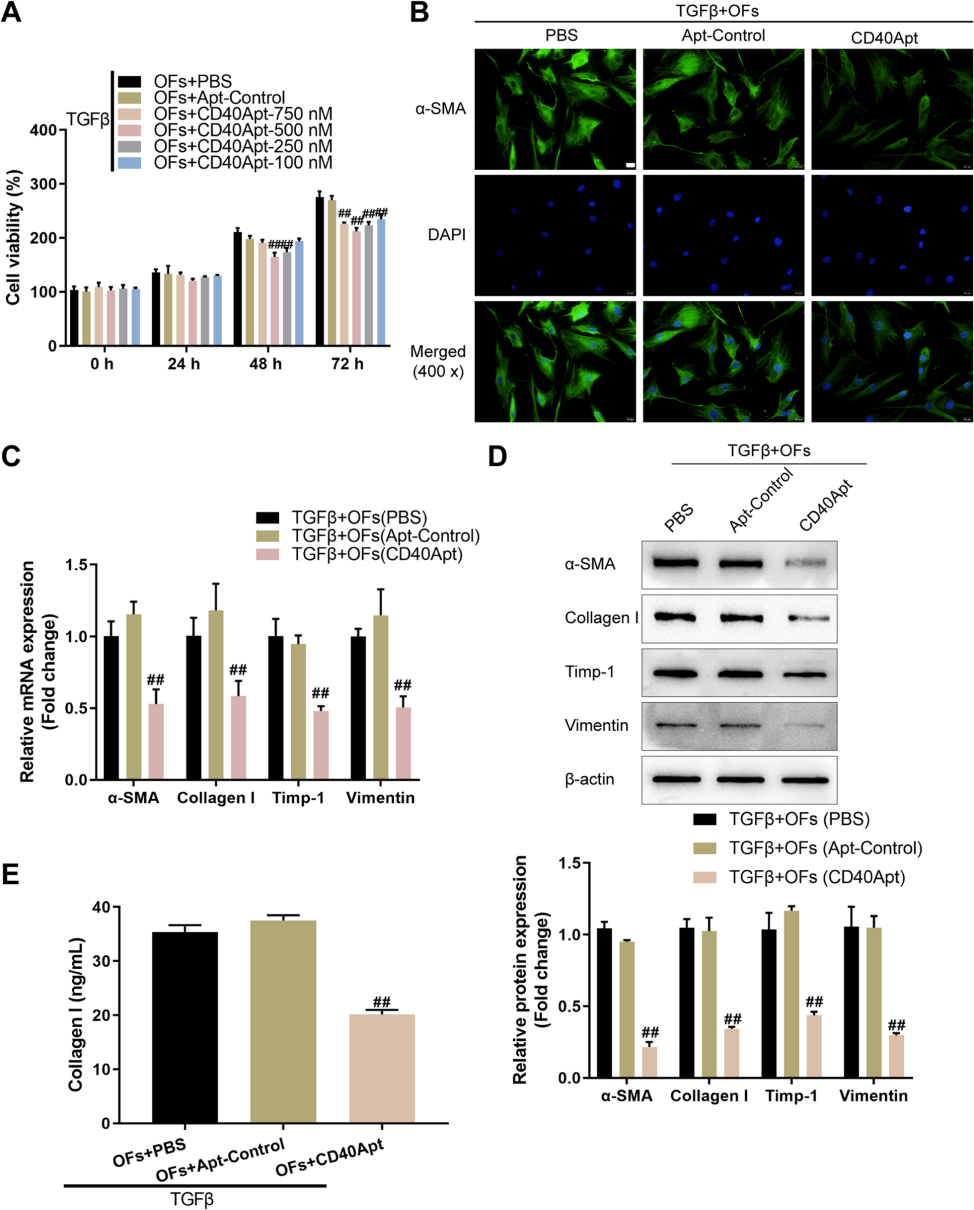
Effects of CD40Apt on TGF-β-induced orbital fibroblast activation. **A** Orbital fibroblasts were challenged with 10 ng/ml TGF-β for 48 h, treated with PBS, non-specific control aptamer, or different concentrations of CD40Apt (750/500/250/100 nM), and examined for cell viability using CCK-8 assay. Then, orbital fibroblasts were challenged with 10 ng/ml TGF-β for 48 h, treated with PBS, non-specific control aptamer, or 500 nM CD40Apt for 48 h, and examined for the levels of α-SMA using Immunofluorescent staining (**B**); the mRNA expression of α-SMA, collagen I, Timp-1, and vimentin using qRT-PCR (**C**); the protein levels of α-SMA, collagen I, Timp-1, and vimentin using Immunoblotting (**D**); the levels of collagen I in supernatant using ELISA (E)

Next, upon TGF-β stimulation, orbital fibroblasts were treated with PBS, non-specific control aptamer, or different concentrations of CD40Apt (750/500/250/100 nM) and examined for cell viability; Fig. 2A shows that TGF-β-induced cell viability could be partially suppressed by all concentrations of CD40Apt at 72 h. Then, orbital fibroblasts were challenged with 10 ng/ml TGF-β for 48 h, treated with PBS, non-specific control aptamer, or 500 nM CD40Apt, and examined for the activation status. CD40Apt administration significantly downregulated α-SMA, collagen I, Timp-1, and vimentin mRNA and protein expression (Fig. 2B–D). Consistently, CD40Apt administration notably inhibited collagen I levels in supernatant (Fig. 2E). These data suggest that CD40Apt administration could partially eliminate TGF-β-induced orbital fibroblast activation.

In order to prove that the function of CD40Apt is achieved by blocking the CD40-CD40L pathway, the effect of CD40Apt on in CD40 knocked-down mouse orbital fibroblasts under TGF-β treatment were investigated (Additional file 3: Fig. S3). Additional file 3: Fig. S3A shows that knockdown of CD40 alone significantly inhibited cell viability, and CD40Apt administration further inhibited cell viability. Next, knockdown of CD40 alone markedly downregulated α-SMA, collagen I, Timp-1, and vimentin mRNA and protein expression, and CD40Apt administration further facilitated the effect of CD40 knockdown on mouse orbital fibroblasts (Additional file 3: Fig. S3B-C). Similarly, knockdown of CD40 alone inhibited collagen I level in supernatant, and CD40Apt administration further inhibited collagen I level (Additional file 3: Fig. S3D). These outcomes concluded that knockdown of CD40 inhibited TGF-β-induced orbital fibroblast activation and CD40Apt further facilitated the effect of CD40 knockdown on orbital fibroblast activation.

**Fig. 3:**
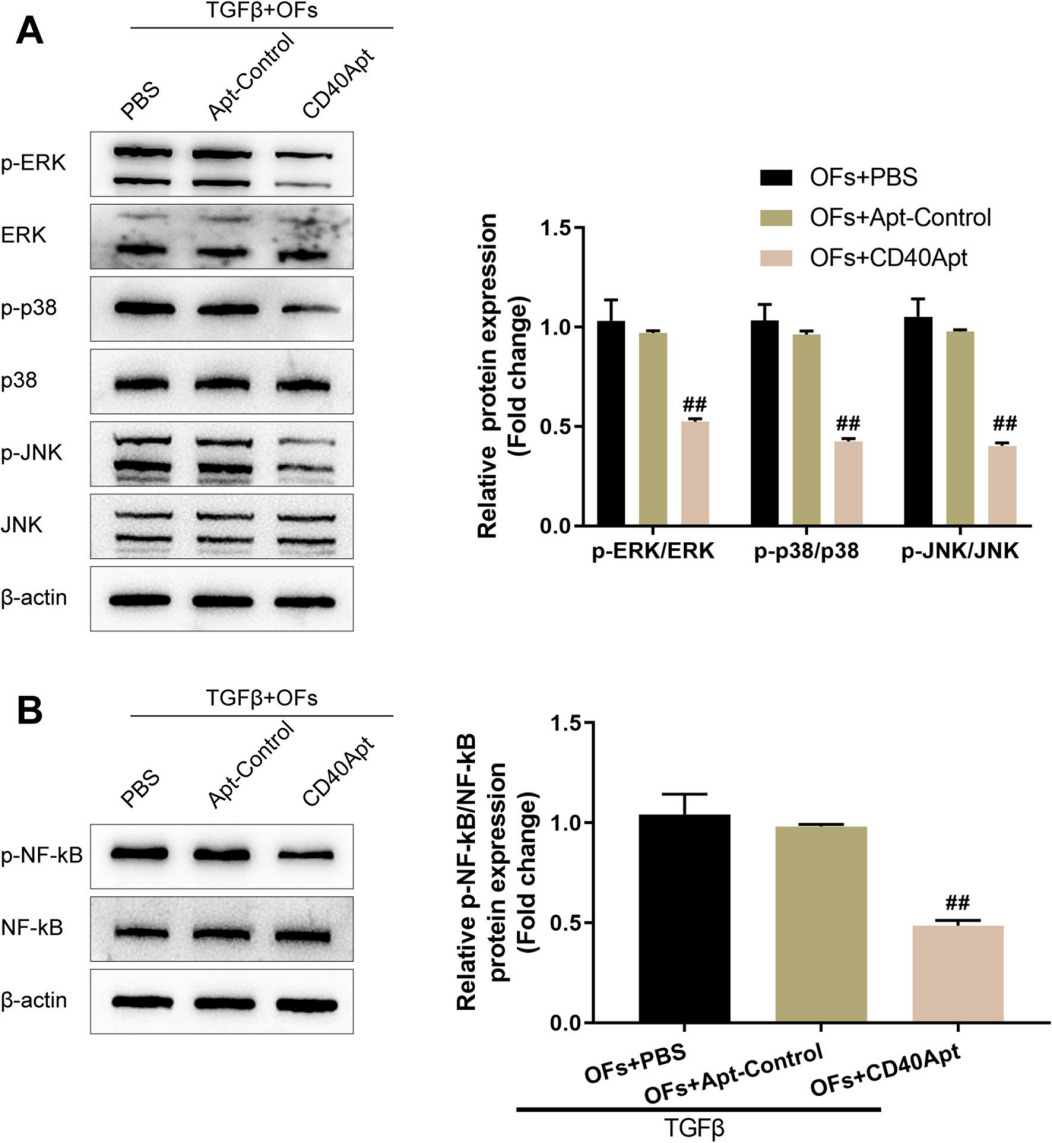
Effects of CD40Apt on the activation of intracellular CD40 and downstream signaling pathways Orbital fibroblasts were challenged with 10 ng/ml TGF-β for 48 h, treated with PBS, non-specific control aptamer, or 500 nM CD40Apt for 48 h, and examined for the protein levels of p-Erk, Erk, p-p38, p38, p-JNK, and JNK using Immunoblotting (**A**); the protein levels of p-NF-κB and NF-κB using Immunoblotting (**B**)

At the same time, the effects of CD40Apt on the activation of intracellular CD40 and downstream signaling pathways were investigated. Mouse orbital fibroblasts were treated accordingly and determined for p-Erk, Erk, p-p38, p38, p-JNK, JNK, p-NF-κB, and NF-κB protein contents using Immunoblotting. Figure 3A, B shows that TGF-β-induced Erk, p38, JNK, and NF-κB phosphorylation was significantly inhibited via CD40Apt, suggesting that CD40Apt could suppress the activation of CD40 and downstream signaling pathways.

### Effects of CD40Apt on TAO mice model

Aptamer stability is important for their effective use in therapeutic or diagnostic applications. Firstly, CD40Apt was incubated with fresh mouse serum to investigate its stability in vitro. The results showed that CD40Apt was stable for 7 days during the incubation period as observed on the agarose gel electrophoresis (Additional file 4: Fig. S4). These outcomes suggested that the CD40Apt with chemical modification has reliable stability and maybe a suitable tool for therapeutic applications.

**Fig. 4:**
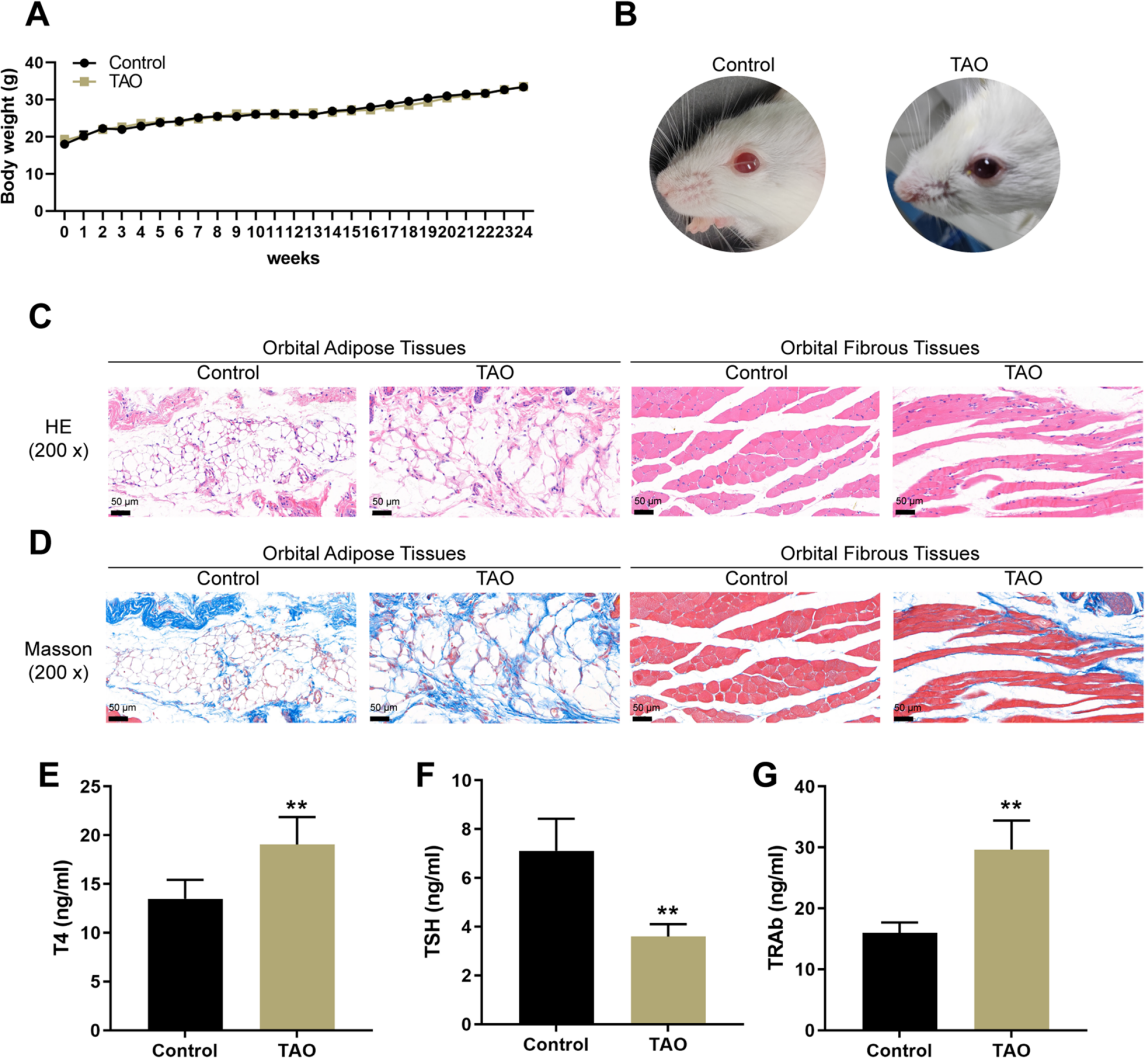
Establishment of TAO model in mice by immunization with TSHR adenovirus. **A** The body weight of mice in the control and model groups every week from week 1 to 24. **B** Representative images of mice (lateral appearances) in different groups. **C**, **D** H&E and Masson staining of orbital adipose and fibrous tissues. **E** Mice serum T4, TSH, and TRAb levels determined using ELISA

**Fig. 5:**
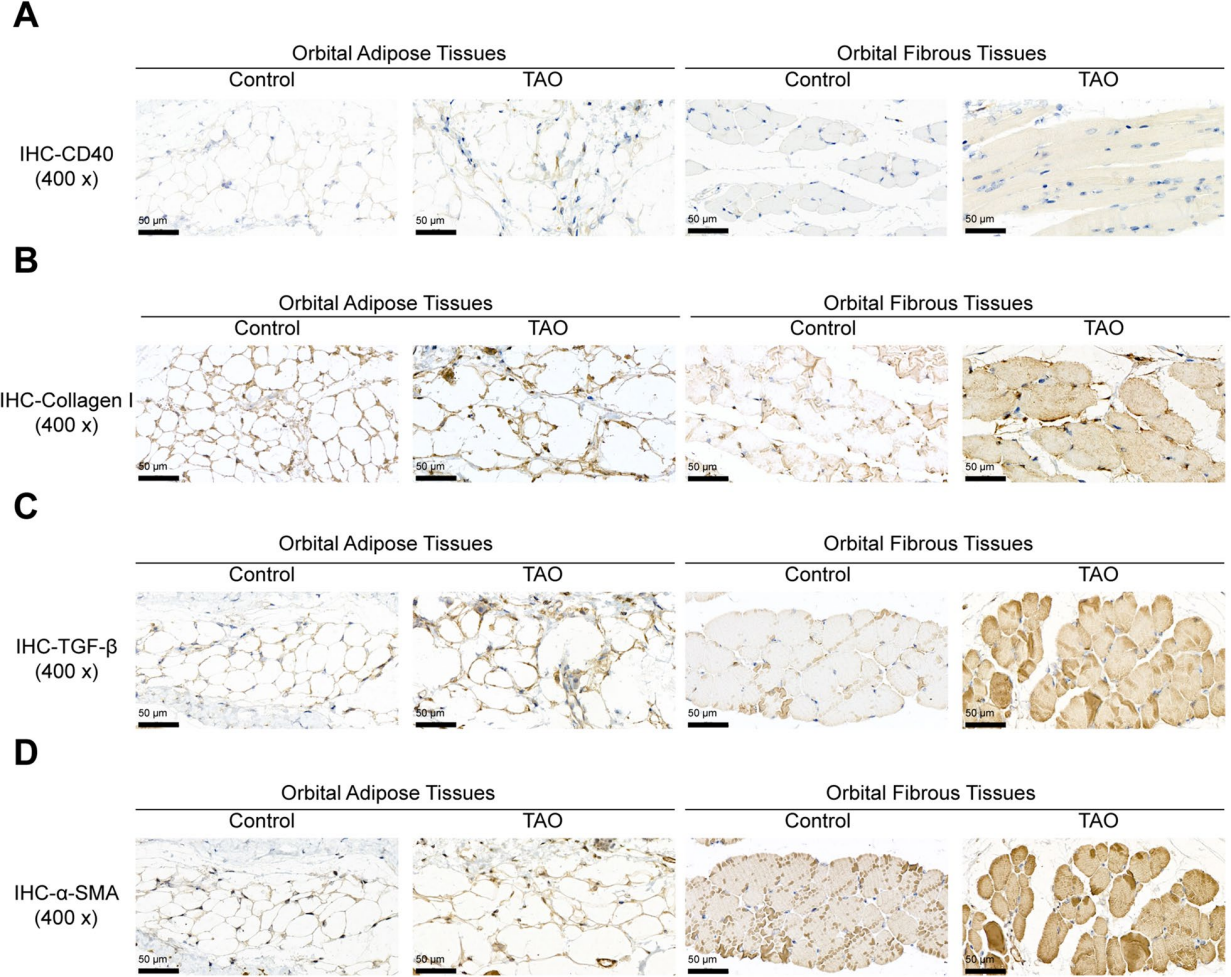
Orbital fibroblast activation and CD40 levels in mice. **A**–**D** The levels of CD40, collagen I, TGF-β, and α-SMA in orbital adipose and fibrous tissues examined using Immunohistochemical staining (IHC)

Next, the effects of CD40Apt on TAO were investigated using mice model. The body weight of mice in the control and model groups was monitored every week from week 1 to 24, and the body weight saw no significant changes in mice from two groups (Fig. 4A). Representative images of mice (lateral appearances) in different groups were shown in Fig. 4B; mice in the Ad-TSHR group showed eyelid broadening, exophthalmos, and conjunctive redness. Histopathological examination on orbital and thyroid tissues shows that compared with the normal group and the Ad-NC group, mice in the Ad-TSHR group had severe inflammatory infiltration among the thyroid glands and between the extraocular muscle space, and the extraocular muscle space was significantly widened with vascular hyperplasia. Moreover, the extraocular muscles in the TSHR-adenovirus group showed to be more hypertrophic compared with normal control (Fig. 4C, D). Consistent with the histopathological changes, the serum T4, and TRAb levels showed to be remarkably elevated, while TSH levels reduced in model mice (Fig. 4E). Regarding the orbital fibroblast activation status, the levels of CD40, collagen I, TGF-β, and α-SMA in orbital muscle and adipose tissues were increased in model mice (Fig. 5A–D), suggesting that orbital fibroblasts were activated in TAO mice.

**Fig. 6:**
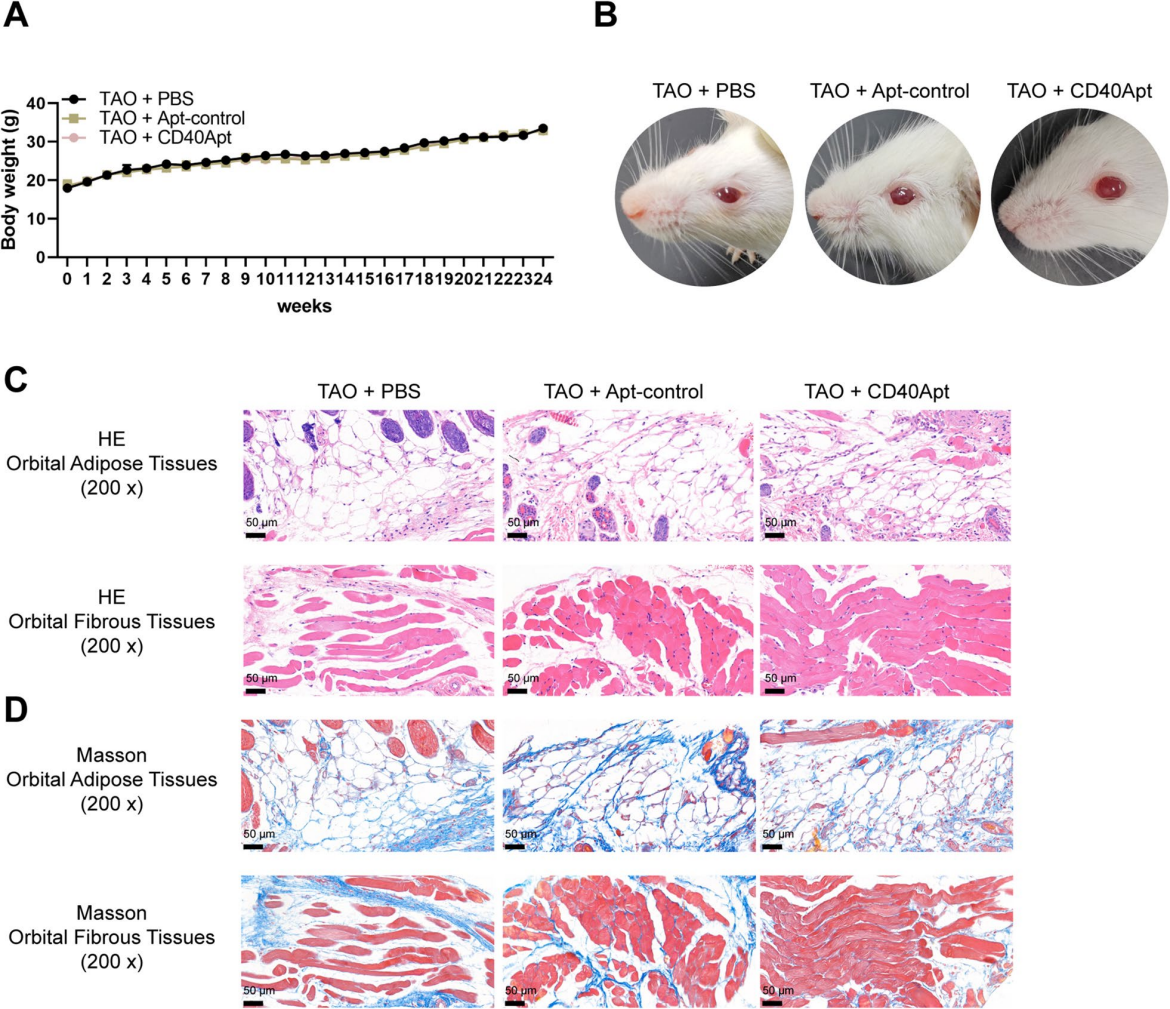
Effects of CD40Apt on TAO mice model Mice were allocated into three groups: TAO + PBS, TAO + non-specific control aptamer, and TAO + CD40Apt; mice in each groups received corresponding modeling and aptamer injection. **A** The body weight of mice in the control and model groups every week from week 1 to 24. **B** Representative images of mice (lateral appearances) in different groups. **C**, **D** H&E and Masson staining of orbital adipose and fibrous tissues

**Fig. 7:**
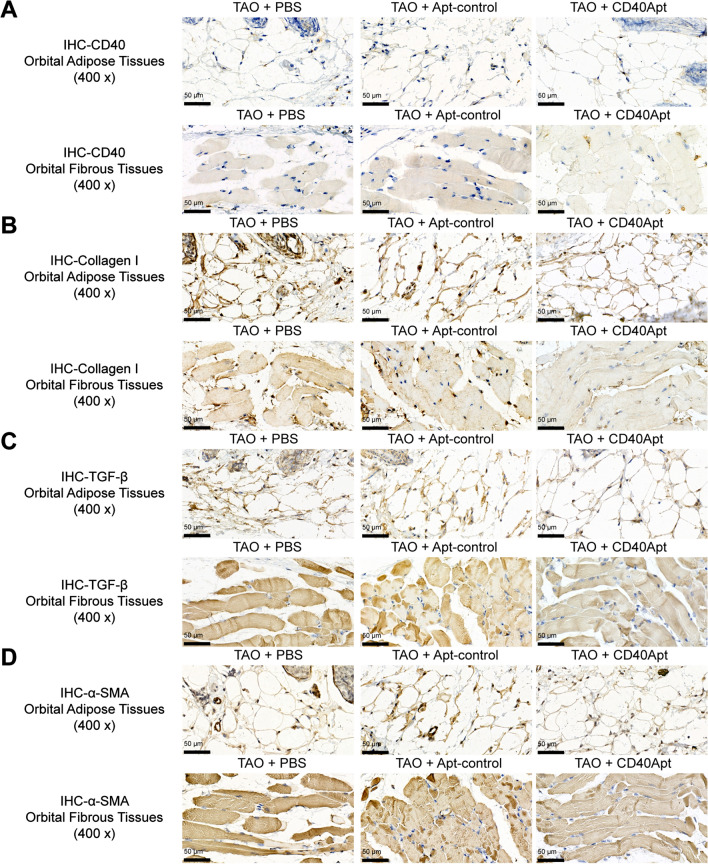
Effects of CD40Apt on orbital fibroblast activation and CD40 levels in mice. **A**–**D**. The levels of CD40, collagen I, TGF-β, and α-SMA in orbital adipose and fibrous tissues examined using IHC

**Fig. 8:**
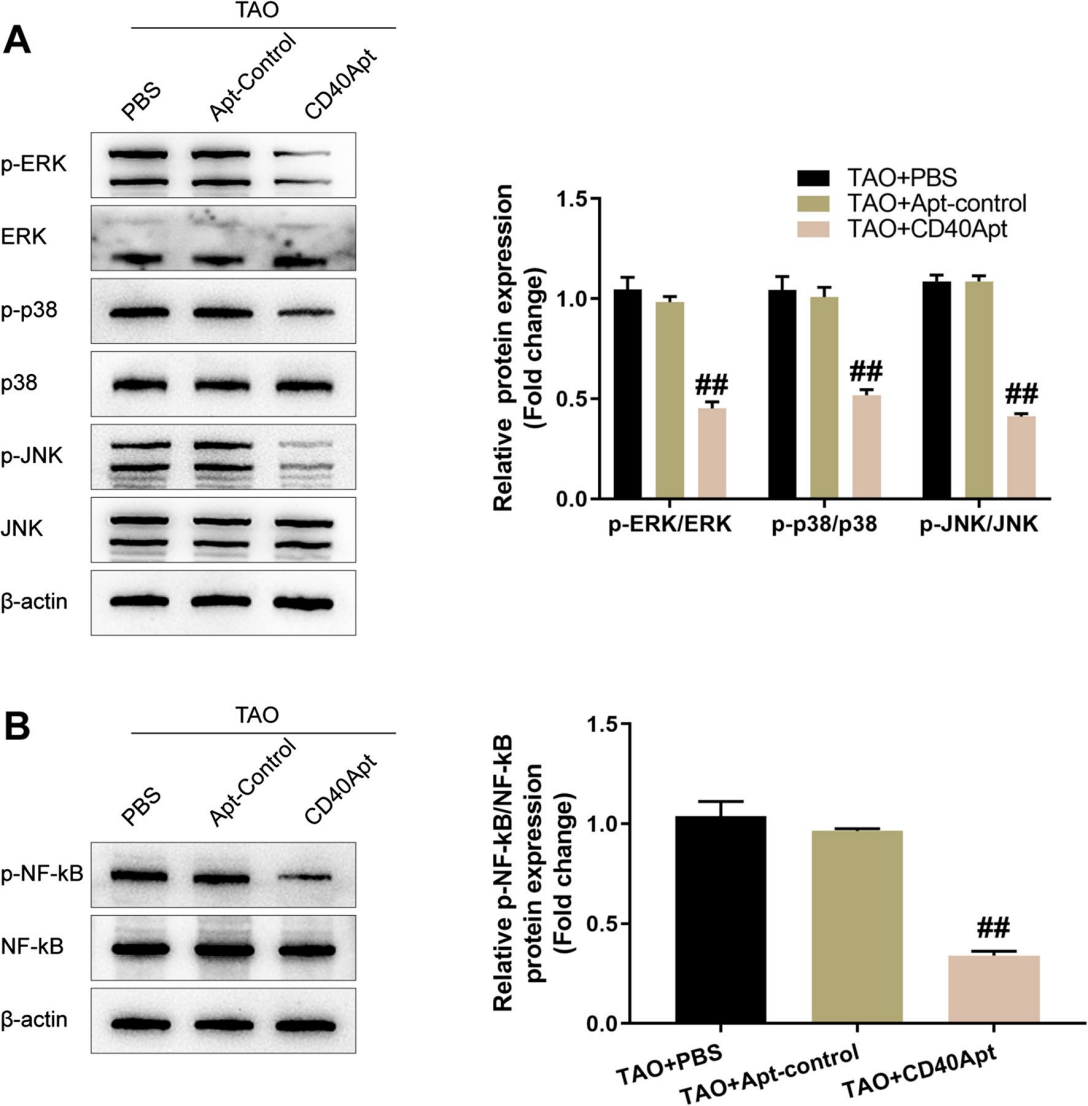
Effects of CD40Apt on the activation of CD40 and downstream signaling pathways in model mice. **A**, **B** The protein levels of p-Erk, Erk, p-p38, p38, p-JNK, JNK, p-NF-κB, and NF-κB in orbital tissues using Immunoblotting

Next, mice were allocated into three groups: TAO + PBS, TAO + non-specific control aptamer, and TAO + CD40Apt; mice in each groups received corresponding modeling and aptamer injection. Figure 6A shows that CD40Apt caused no significant differences to the body weight of mice as compared to the control group and non-specific aptamer group. The representative images of mice (lateral appearances) shows that CD40Apt partially improved the eyelid broadening, exophthalmos, and conjunctive redness (Fig. 6B). H&E and Masson staining of orbital muscle and adipose tissues shows that CD40Apt ameliorated inflammatory infiltration and the hyperplasia in orbital muscle and adipose tissues (Fig. 6C, D). Concerning orbital fibroblast activation, CD40Apt reduced the levels of CD40, collagen I, TGF-β, and α-SMA in orbital muscle and adipose tissues of model mice (Fig. 7A–D). Finally, regarding the CD40 and downstream signaling pathways, CD40Apt administration significantly suppressed Erk, p38, JNK, and NF-κB phosphorylation (Fig. 8A, B).

## Discussion

In this study, CD40Apt was confirmed to specifically recognize CD40-positive cells and selectively bind to CD40 protein from mouse orbital fibroblasts. In TGF-β-induced orbital fibroblast activation model in vitro, CD40Apt administration inhibited TGF-β-induced cell viability, decreased TGF-β-induced α-SMA, Collagen I, Timp-1, and vimentin levels, and suppressed TGF-β-induced phosphorylation of Erk, p38, JNK, and NF-κB. In TAO mice model in vivo, CD40Apt caused no significant differences to the body weight of mice; furthermore, CD40Apt improved the eyelid broadening, ameliorated inflammatory infiltration and the hyperplasia in orbital muscle and adipose tissues in model mice. Concerning orbital fibroblast activation, CD40Apt reduced the levels of CD40, collagen I, TGF-β, and α-SMA in orbital muscle and adipose tissues of model mice. Finally, CD40Apt administration significantly suppressed Erk, p38, JNK, and NF-κB phosphorylation.

The CD40-CD40L signaling contributes to TAO progression through promoting inflammatory factor release and hyaluronic acid synthesis by orbital fibroblasts [14–16]. Hyaluronic acid could accumulate in the orbital tissues and infiltrate TAO patients’ extraocular muscles [4]. In this study, TGF-β-stimulated mouse orbital fibroblasts expressed higher extracellular matrix molecules including α-SMA, collagen I, Timp-1, and vimentin, whereas CD40Apt significantly decreased the levels of these factors. Furthermore, CD40 and downstream Erk, p38, JNK, and NF-κB pathways were activated in TGF-β-stimulated orbital fibroblasts, whereas inhibited by CD40Apt. As aforementioned, CD40 can activate a number of signaling pathways, culminating in the NF-κB recruitment into the nucleus [27]. A genetic investigation has connected CD40 and NF-κB pathway with rheumatoid arthritis, indicating that both molecules play a critical role [28]. The findings in this study also suggest that CD40Apt inhibits TGF-β-induced orbital fibroblast activation through suppressing cell viability and decreasing the synthesis of extracellular matrix molecules, consistent with previous studies [29].

Transfected fibroblasts, as well as plasmid or adenoviral vaccinations containing extracellular human TSHR, were used. A subunit plasmid was utilized to mimic human Graves' disease in mice [30]. For example, adenovirus-induced immunization with the A domain of TSHR (Ad-TSHR) in BALB/c mice was successfully induced to establish a mouse model of TAO [31–33]. In this study, model mice had expanded eyelids, exophthalmia and conjunctive redness, significant inflammatory infiltration between the extraocular muscle space, and hypertrophic extraocular muscles and adipose tissues. Furthermore, mean T4 levels showed to be consistently and considerably increased in Ad-TSHR-immunized mice compared with normal control [34]. In this study, raised serum T4 and TRAb levels and downregulated TSH were observed in model mice, suggesting the successful establishment of the TAO model in mice. Concerning orbital fibroblast activation, elevated α-SMA, collagen I, Timp-1, and vimentin were observed, suggesting that orbital fibroblasts were activated in TAO mice. Consistent with in vitro observations, CD40Apt administration in mice model significantly improved the histopathological changes, including inflammatory infiltration and hypertrophic extraocular muscles and adipose tissues. Notably, the synthesis of extracellular matrix molecules was also suppressed by CD40Apt. Moreover, CD40Apt administration significantly suppressed Erk, p38, JNK, and NF-κB phosphorylation in model mice, suggesting that CD40Apt improves TAO in model mice through inhibiting orbital fibroblast activation via the CD40 and downstream signaling pathways.

In conclusion, CD40Apt could specifically bind to CD40 proteins in their natural state on the cell surface with high affinity. Through the CD40 and downstream signaling pathways, CD40Apt suppresses orbital fibroblast activation, therefore improving TAO in mice model. CD40Apt represents a promising antagonist of the CD40-CD40L signaling for TAO treatment. However, in this study, only aptamers targeting mouse CD40 were used for in vivo and in vitro studies, the screening and application of aptamers against human CD40 needs further study in the future.

## Supplementary Information

**Additional file 1: Figure S1.** Schematic diagram of animal experimental treatment process

**Additional file 2: Figure S2.** Orbital fibroblast activation model by TGF-β challenge. Normal mouse and and TAO mouse isolated orbital fibroblasts (NOFs and OFs) were examined for the levels of α-SMA using Immunofluorescent staining (A). The TAO orbital fibroblasts were stimulated with 10 ng/ml TGF-β for 48 h and examined for cell viability by CCK-8 assay (B); the mRNA expression of α-SMA, collagen I, Timp-1, and vimentin using qRT-PCR (C); the protein levels of α-SMA, collagen I, Timp-1, and vimentin using Immunoblotting (D); the levels of collagen I in supernatant using ELISA (E).

**Additional file 3: Figure S3.** Effects of CD40 knockdown and CD40Apt on TGF-β-induced orbital fibroblast activation Orbital fibroblasts were transfected with sh-NC and sh-CD40 vector and then treated with 10 ng/ml TGF-β, non-specific control aptamer, or 500 nM CD40Apt for 48 h, and examined for (A) cell viability using CCK-8 assay; (B) the mRNA expression of α-SMA, collagen I, Timp-1, and vimentin using qRT-PCR; (C) the protein levels of α-SMA, collagen I, Timp-1, and vimentin using Immunoblotting; (D) the levels of collagen I in supernatant using ELISA.

**Additional file 4: Figure S4.** Serum stability of CD40Apt. Gel electrophoresis shows the stability of CD40Apt in fresh mouse serum over 7 days of incubation.

## Data Availability

Please contact the corresponding author for data requests.
