# Supplementary figures and images for: RNA aptamers with specific binding affinity to CD40 (CD40Apt) represents a promising antagonist of the CD40-CD40L signaling for thyroid-associated ophthalmopathy (TAO) treatment in mouse
Source: J Transl Med. 2023 Jun 18;21:396. doi: 10.1186/s12967-023-04217-0 (PMC10278284; doi:10.1186/s12967-023-04217-0)

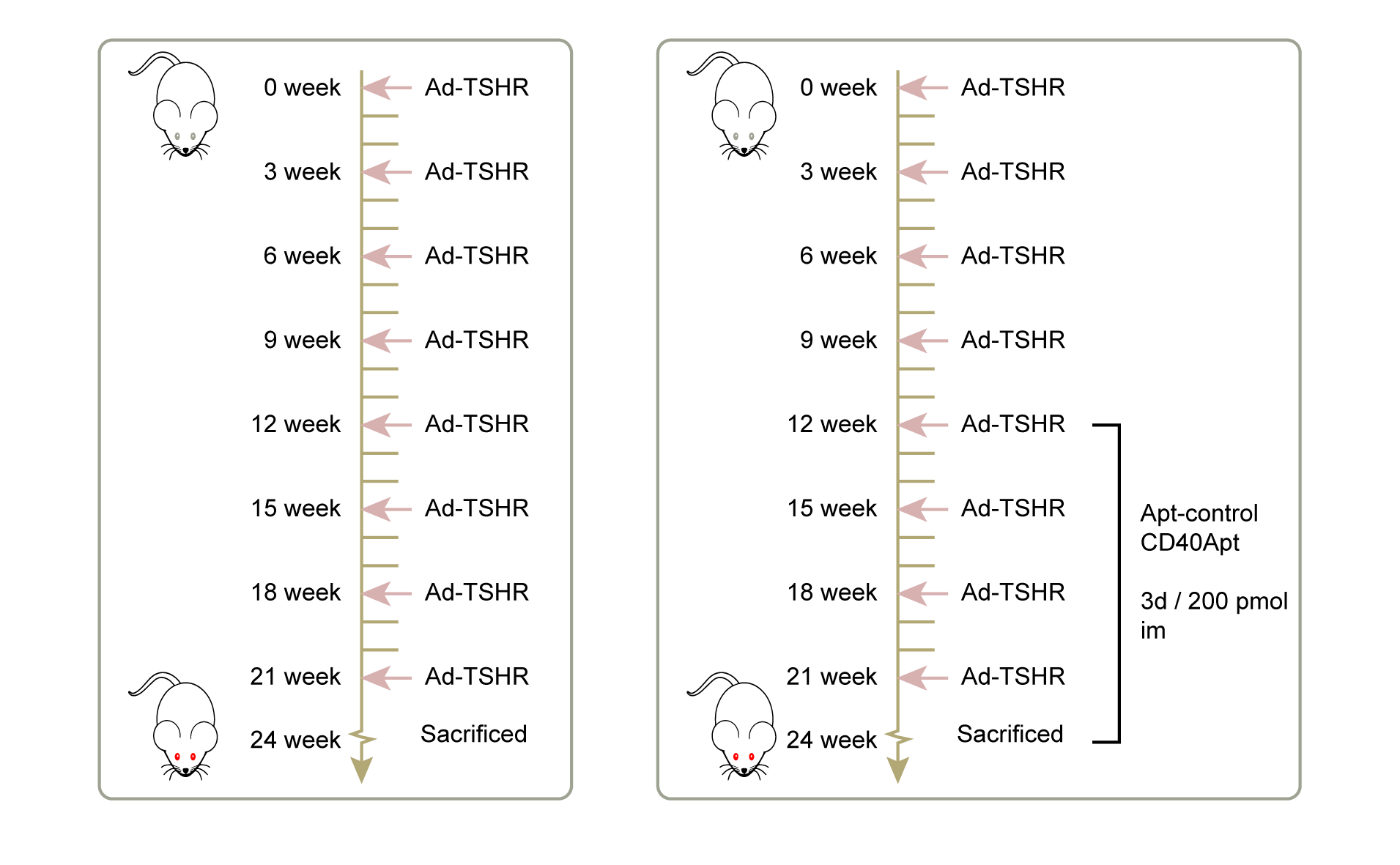

Supplement: Supplementary file 1 — Additional file 1: Figure S1. Schematic diagram of animal experimental treatment process [file 12967_2023_4217_MOESM1_ESM.tif]

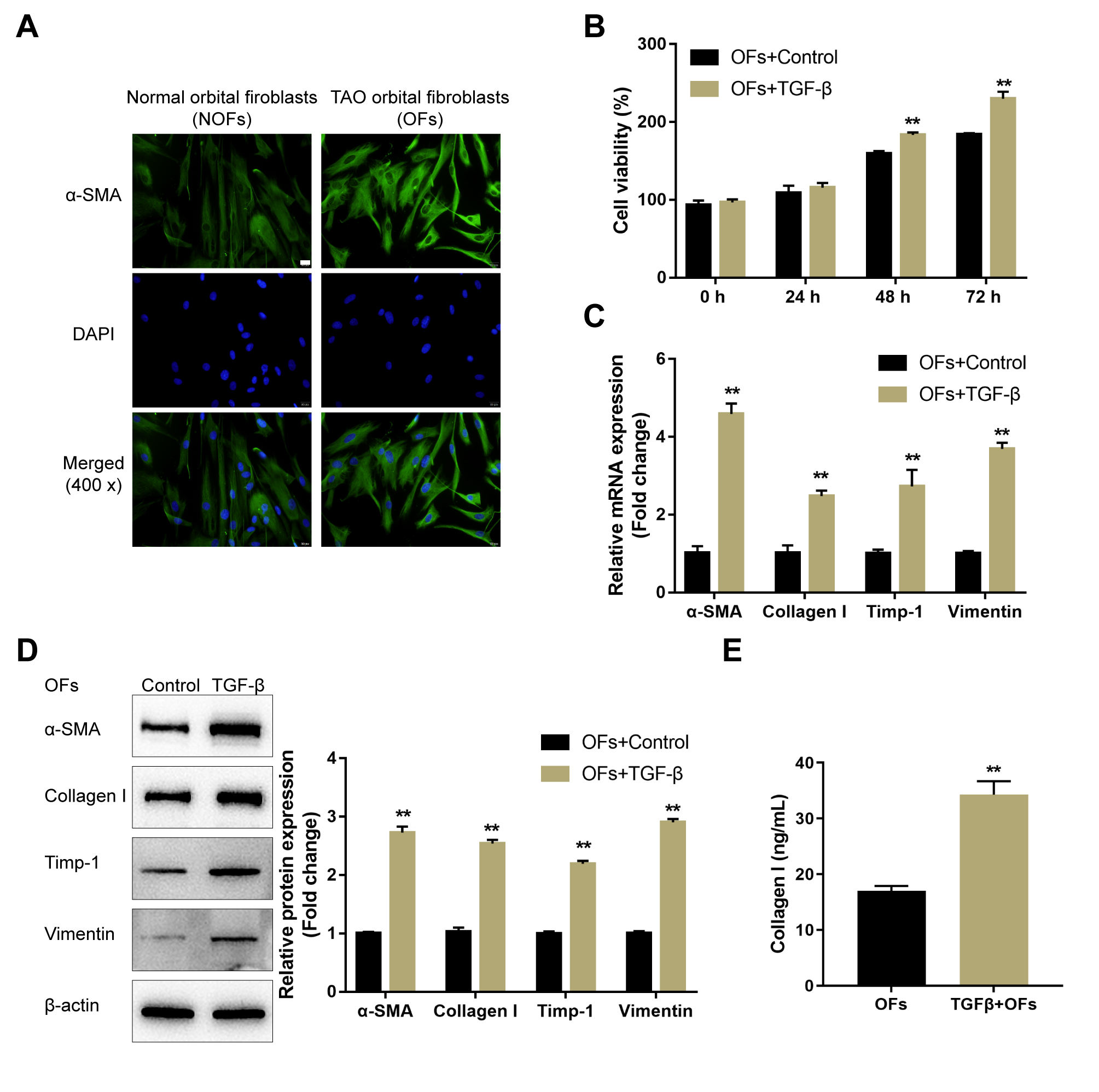

Supplement: Supplementary file 2 — Additional file 2: Figure S2. Orbital fibroblast activation model by TGF-β challenge. Normal mouse and and TAO mouse isolated orbital fibroblasts (NOFs and OFs) were examined for the levels of α-SMA using Immunofluorescent staining (A). The TAO orbital fibroblasts were stimulated with 10 ng/ml TGF-β for 48 h and examined for cell viability by CCK-8 assay (B); the mRNA expression of α-SMA, collagen I, Timp-1, and vimentin using qRT-PCR (C); the protein levels of α-SMA, collagen I, Timp-1, and vimentin using Immunoblotting (D); the levels of collagen I in supernatant using ELISA (E). [file 12967_2023_4217_MOESM2_ESM.tif]

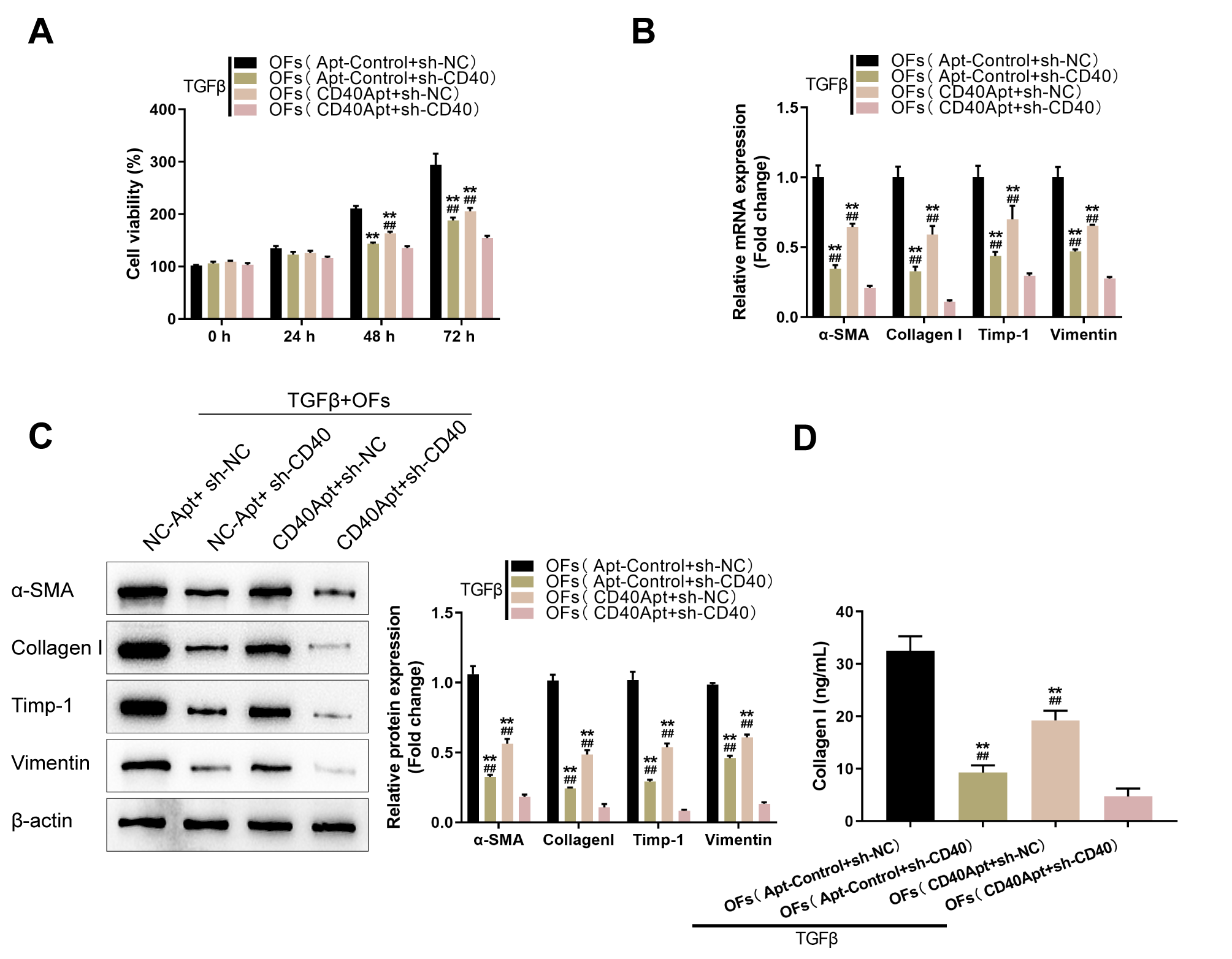

Supplement: Supplementary file 3 — Additional file 3: Figure S3. Effects of CD40 knockdown and CD40Apt on TGF-β-induced orbital fibroblast activation Orbital fibroblasts were transfected with sh-NC and sh-CD40 vector and then treated with 10 ng/ml TGF-β, non-specific control aptamer, or 500 nM CD40Apt for 48 h, and examined for (A) cell viability using CCK-8 assay; (B) the mRNA expression of α-SMA, collagen I, Timp-1, and vimentin using qRT-PCR; (C) the protein levels of α-SMA, collagen I, Timp-1, and vimentin using Immunoblotting; (D) the levels of collagen I in supernatant using ELISA. [file 12967_2023_4217_MOESM3_ESM.tif]

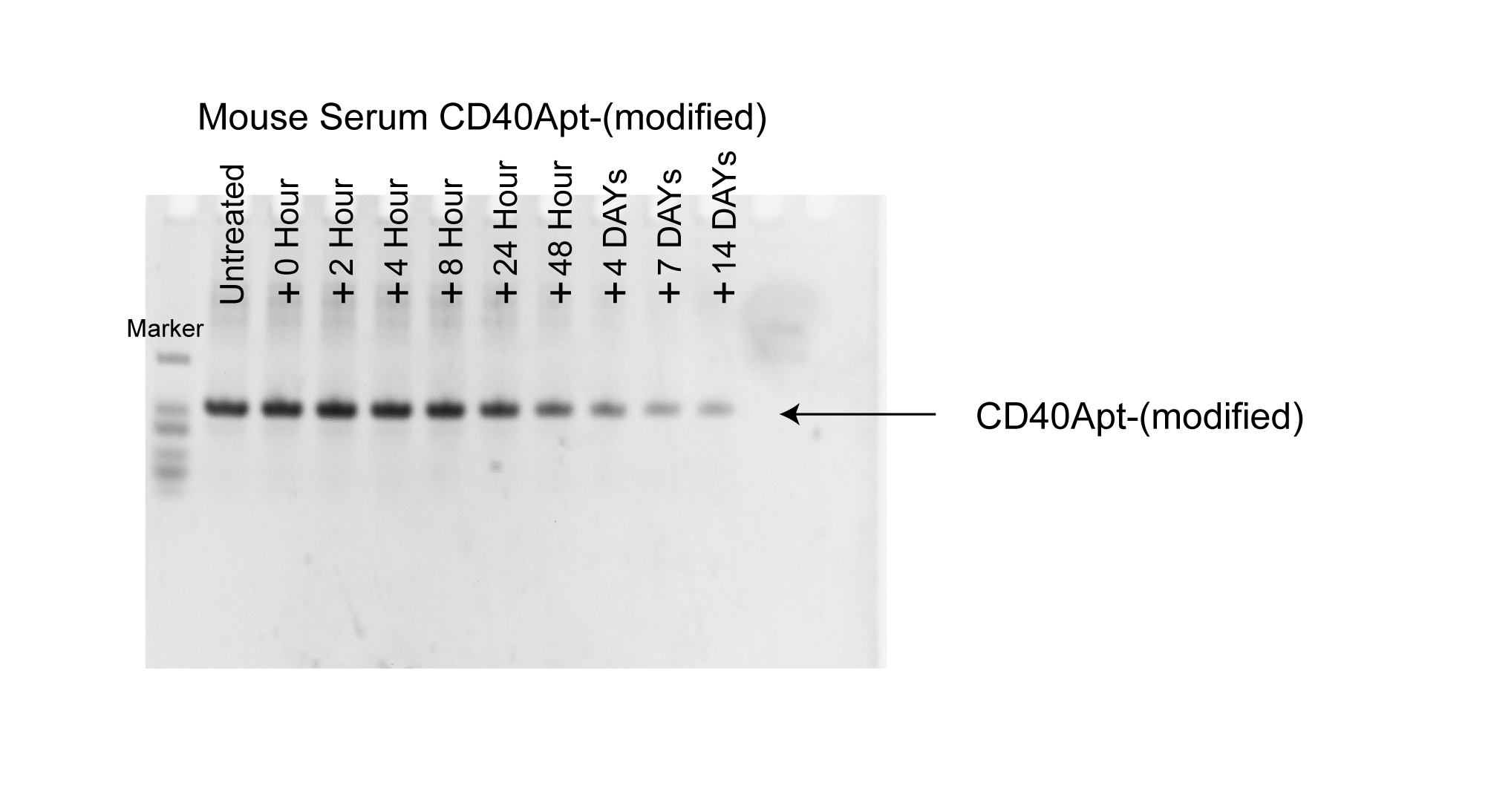

Supplement: Supplementary file 4 — Additional file 4: Figure S4. Serum stability of CD40Apt. Gel electrophoresis shows the stability of CD40Apt in fresh mouse serum over 7 days of incubation. [file 12967_2023_4217_MOESM4_ESM.tif]
